# A Literature Review on Holistic Well-Being and Dopamine Fasting: An Integrated Approach

**DOI:** 10.7759/cureus.61643

**Published:** 2024-06-04

**Authors:** Dev Desai, Jekee Patel, Falak Saiyed, Himarshi Upadhyay, Prashant Kariya, Jitendra Patel

**Affiliations:** 1 Internal Medicine, Smt. Nathiba Hargovandas Lakhmichand Municipal Medical College, Ahmedabad, IND; 2 Surgery, Gujarat Medical Education and Research Society Medical College, Vadnagar, IND; 3 Medicine, Gujarat Medical Education and Research Society Medical College, Vadnagar, IND; 4 Pediatrics, Kiran Medical College, Surat, IND; 5 Physiology, Gujarat Medical Education and Research Society Medical College, Vadnagar, IND

**Keywords:** holistic approach, self-care, mindfulness, resilience, detoxification, dopamine fasting

## Abstract

Popularly known as dopaminergic detox or dopamine fasting, it is a concept that aims at reducing dependence on instant satisfaction gratification and overstimulation to attain mental clarity, lessen anxiety, and be able to enjoy everyday events again. Digital detox has been a part of the dopamine fasting concept for several years now. However, some critics argue that this notion has no scientific proof behind it and may fail to deal with the problem of dopamine dysregulation. Some intense types of dopamine fasting which include extreme isolation or strict dieting can result in damage to mental health as well as physical fitness. The objective of the article is to understand what dopamine fasting means and see the literature and evidence available on the topic. Indexes like PubMed, Scopus, OVID, Embase, and Google Scholar were searched using the keywords to understand the existing knowledge about dopamine fasting. The literature review was then written to incorporate the understanding in a way that can be implemented practically. Recent studies have shown that individuals who engage in dopamine-fasting-like ideologies may experience reduced impulsive behaviors, increased focus on tasks, and reduced overwhelm. However, extreme forms of dopamine fasting can lead to feelings of loneliness, anxiety, and malnutrition, which can have detrimental effects on mental and physical health. Hence, the effects of dopamine fasting can vary greatly among individuals, and there is no one-size-fits-all approach. It is essential to consider individual needs and preferences when incorporating dopamine fasting into one’s lifestyle and explore alternative practices that align with the principles of dopamine fasting. Understanding and respecting these differences is crucial in determining the most suitable strategies for maintaining a balanced dopamine response and overall psychological health. The benefits of dopamine fasting can be tremendous if done correctly but it depends on every individual to find the correct way and in the modern day, the practices can become tough to implement.

## Introduction and background

Dopamine fasting, also known as dopaminergic fasting or dopamine detox, is a concept that has gained popularity in recent years. Proponents of dopamine fasting claim that it can help individuals reduce their reliance on instant gratification and overstimulation, leading to a greater sense of control and focus in their lives. The practice involves abstaining from certain stimuli, such as electronic devices, social interaction, and even food, to allow the brain to reset and recalibrate its dopamine response [1,2].

There is evidence to suggest that excessive dopamine stimulation from activities like social media, video games, and junk food can lead to desensitization of the brain’s reward system, ultimately contributing to issues like addiction, impulsivity, and difficulty in maintaining attention [3-6]. By taking a break from these stimuli, individuals may experience improved mental clarity, reduced anxiety, and a renewed ability to find pleasure in simple, everyday activities [7]. However, critics of dopamine fasting argue that the concept lacks scientific backing and may not be an effective approach to addressing issues related to dopamine dysregulation. There are concerns that extreme forms of dopamine fasting, such as prolonged periods of isolation or severe dietary restrictions, could have negative implications for mental and physical health [8,9].

As research on dopamine fasting continues to emerge, it is important to weigh the potential benefits against the possible disadvantages and harm to the body. The nuanced nature of this topic warrants further exploration and consideration of individual circumstances to determine whether dopamine fasting is a suitable practice for promoting overall well-being and psychological balance. Recent studies have shed light on some potential benefits of dopamine fasting, supporting the claims of its proponents. Research has indicated that individuals who engage in dopamine fasting may experience a reduction in impulsive behaviors and an increased ability to focus on tasks for longer periods [10,11]. Additionally, some individuals have reported feeling less overwhelmed and more in control of their thoughts and actions after implementing regular dopamine fasting practices into their routines. On the other hand, it is crucial to acknowledge the potential disadvantages and harm that extreme forms of dopamine fasting can pose to the body [12,13]. Prolonged isolation and severe dietary restrictions can lead to feelings of loneliness, anxiety, and malnutrition, which can have detrimental effects on mental and physical health. It is imperative to approach dopamine fasting with moderation and to prioritize overall well-being while considering its implementation [14].

Dopamine fasting is meant to create an ideology of mindfulness about self and the surroundings and being with the one. Although a new ideology, it dates back to old Indian and Chinese philosophies adopted by Sadhus, who would give up everything just to focus on being with nature and being part of it. The process of being selfless along with having control over emotions and lustful things is embedded in the culture of these countries [15]. Emotional connection with self, nature, and others creates the boundaries of mindfulness [15].

The framework of dopamine fasting not only means to give away everything you desire but it is a guide toward a more insightful life. From being more mentally resilient to developing a connection with the surroundings and leading a peaceful life is a path that can lead to eternal calmness and happiness. The generations thousands of years ago used to indulge in practices that looked arbitrary and absurd to a modern-day abstract thinker but those practices had an impact, as the current generations are one of the worst psychiatric disease-affected populations recorded in human history even with all the biases [15].

As the concept of dopamine fasting continues to evolve, it is essential for individuals to critically evaluate its applicability to their own lives. Engaging in open discussions and seeking guidance from healthcare professionals can provide valuable insights into whether dopamine fasting is a suitable practice for promoting psychological balance and overall well-being [16]. Exploring alternative practices that align with the principles of dopamine fasting may also be beneficial. Mindfulness activities, such as meditation and yoga, have been shown to have positive effects on dopamine regulation and mental well-being [17,18]. These practices provide an opportunity to unplug from digital distractions and cultivate a greater awareness of the present moment, without the extreme measures associated with dopamine fasting [19,20].

As every human individual is different from others, the implementation of the ideology becomes different. Each person requires an individual set of boundaries and directions to achieve dopamine fasting and even though some generalizations can be made according to epidemiological or population-based knowledge, those might not be enough [21]. The goals as mentioned further are the ones that need to be achieved for any person wishing to adopt dopamine fasting as an ideology and implement it in life [22-24].

It is important to recognize that the effects of dopamine fasting can vary greatly among individuals, and there is no one-size-fits-all approach. Some people may find it beneficial in moderation, while others may not resonate with the concept at all. Understanding and respecting these differences is paramount in determining the most suitable strategies for maintaining a balanced dopamine response and overall psychological health [18]. As individuals navigate their journey with dopamine fasting, it is important to remain open to alternative strategies and interventions that may contribute to psychological balance and well-being. Seeking professional guidance from healthcare providers or mental health professionals can provide personalized recommendations and support in developing an approach that aligns with individual needs and goals [14,25]

The objectives of the study are to understand the current knowledge, literature, and existing evidence on dopamine fasting and to summarize and possibly show a practical way for modern-day individuals to implement the ideology to lead a more insightful and fulfilling life

In essence, the concept of dopamine fasting continues to spark discussions and considerations within the realm of psychological well-being. Striking a balance between exploring innovative practices while prioritizing holistic health remains pivotal in individuals’ pursuit of psychological equilibrium. While dopamine fasting may offer potential benefits, its integration into one’s lifestyle should be approached with careful deliberation and alignment with overall well-being goals.

## Review

The role of dopamine fasting in modern well-being

Understanding the role of dopamine in everyday life contributes to a comprehensive approach to well-being. While dopamine fasting aims to modulate dopamine levels, it is essential to recognize that dopamine plays a crucial role in motivation, reward processing, and cognitive function [2,6,10]. Acknowledging this multifaceted role can lead to a more nuanced understanding of dopamine fasting and its potential implications for individual well-being. Placing the concept of dopamine fasting within the broader context of modern well-being prompts a vital consideration of its potential impact on individual psychological balance. As individuals navigate the evolving landscape of well-being practices, a holistic and individualized approach that considers both the potential benefits and drawbacks of dopamine fasting is essential. By remaining open to diverse strategies and seeking professional guidance, individuals can optimize their well-being while fostering a broader understanding of the complex interplay between neurological processes and lifestyle choices.

Current evidence on dopamine fasting

A scientifically new topic recently taking shape has not been studied much and with proper technique. Dopamine fasting has been a practice for ages but scientifically, it has not been proven yet. The logical abstract mind may accept the idea behind the entire subject as sound, but that does not create an opportunity as a treatment protocol for diseases, especially psychiatric diseases. This lack of reward has been paramount in handicapping research into dopamine fasting and other associated ideologies or techniques that can be useful if not in treating patients than as a prevention measure.

Understanding the nuances of dopamine fasting

Exploring the nuanced nature of dopamine fasting involves acknowledging the varying responses among individuals. While some may find it beneficial in moderation, it is equally important to recognize that others may not resonate with the concept at all. Understanding and respecting these differences is paramount in determining the most suitable strategies for maintaining a balanced dopamine response and overall psychological health [9]. To further enhance the pursuit of psychological equilibrium, integrating holistic practices into one’s daily routine can complement the principles of dopamine fasting. Mindfulness activities, such as meditation and yoga, have demonstrated positive effects on dopamine regulation and mental well-being [16,18]. These practices offer an opportunity to unplug from digital distractions and cultivate a greater awareness of the present moment without the extreme measures associated with dopamine fasting. Striking a balance between exploring innovative practices and prioritizing holistic health remains pivotal in individuals’ pursuit of psychological equilibrium. While dopamine fasting may offer potential benefits, its integration into one’s lifestyle should be approached with careful deliberation and alignment with overall well-being goals [11]. Embracing personalized well-being practices entails recognizing the unique nature of individual responses to dopamine fasting. By adopting a personalized approach, individuals can tailor their well-being strategies to align with their specific needs and preferences. This customization may involve experimenting with different techniques and observing how they influence one's psychological balance, thereby fostering a sense of agency and empowerment in the pursuit of well-being [12].

**Figure 1:**
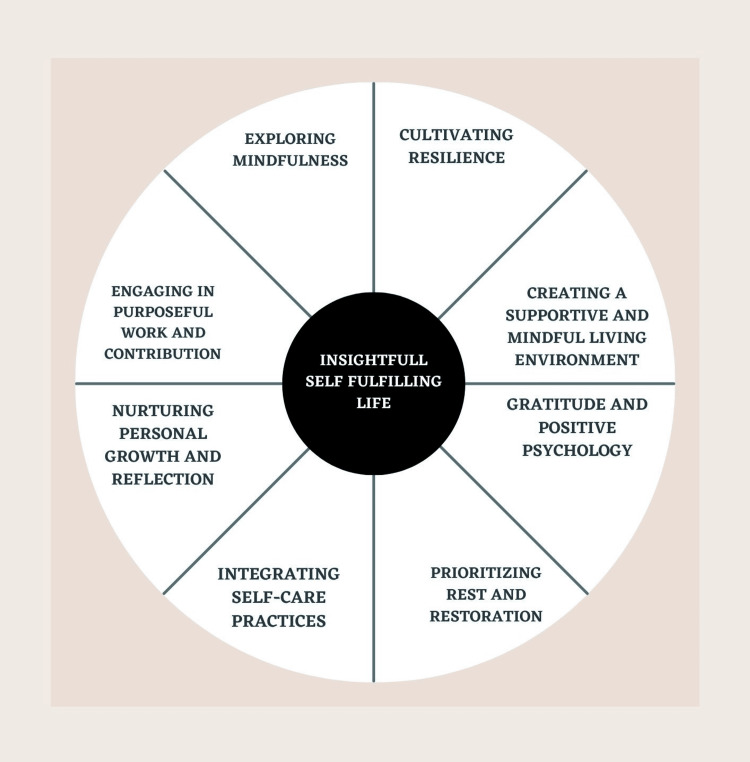
Route to a meaningful and gratifying life

Dopamine fasting when included with the concepts depicted in the figure given below and discussed further can lead to salvation and entire sanctification (Figure 1).

Cultivating Resilience

Incorporating resilience-building practices and mindful awareness can further augment the pursuit of psychological balance [26]. Cultivating resilience involves developing coping strategies to navigate challenges and setbacks, and promoting emotional well-being in the face of stressors. Additionally, mindful awareness practices, such as deep breathing exercises and grounding techniques, can enhance present-moment focus and emotional regulation without necessitating complete withdrawal from dopamine-inducing activities [1,2,5,18,19]. Recognizing the importance of resilience in navigating life’s challenges is a fundamental aspect of holistic well-being. Cultivating resilience involves developing coping mechanisms, embracing adversity as an opportunity for growth, and fostering a mindset of perseverance. By cultivating resilience, individuals strengthen their ability to navigate the complexities of life with fortitude and adaptability [27]. Incorporating these dimensions of holistic well-being into your personalized practices promotes a comprehensive approach that encompasses physical, emotional, and social well-being. By embracing the interconnectedness of these elements, you can further enrich your journey toward holistic well-being and cultivate a balanced and fulfilling life [28]. The most important part of well-being is to have a combination of intellectual growth and experience. Engaging in continuous learning endeavors nurtures intellectual stimulation and a deeper understanding of the complex interplay between brain function and overall well-being. Whether it is through reading, attending educational seminars, or learning new skills, the pursuit of knowledge contributes to a sense of purpose and fulfillment [26].

Gratitude and Positive Psychology

Cultivating gratitude and integrating principles of positive psychology into daily life can significantly contribute to a well-being approach. Engaging in practices that acknowledge and appreciate the positive aspects of life fosters a mindset of abundance and resilience. Additionally, embracing positive psychology principles, including cultivating strengths and virtues, can empower individuals to navigate life’s complexities with a constructive and optimistic outlook. This mindset might become a key to dopamine fasting and mental well-being. It promotes a comprehensive and adaptive approach that acknowledges the multifaceted nature of individual well-being, inviting a continual exploration and refinement of strategies for achieving a balanced and fulfilling life. As you continue to explore holistic well-being, consider incorporating practices that encompass physical, emotional, and spiritual dimensions. Engaging in activities such as yoga, tai chi, or mindfulness-based stress reduction can foster a harmonious balance that supports overall wellness [29]. These practices facilitate the integration of mind-body connections, aligning with the holistic principles of dopamine fasting and promoting a sense of inner harmony. Recognizing the significance of supportive relationships and community ties can significantly impact psychological well-being. Engaging in meaningful connections and seeking support from like-minded individuals can foster a sense of belonging and emotional fulfillment. By nurturing these relationships, individuals can cultivate a supportive environment that complements their well-being journey while offering valuable insights and encouragement [30].

Integrating Self-Care Practices

Self-care practices, including sleep, nutrition, and physical activity, are crucial for promoting overall well-being. These practices align with dopamine fasting principles, promoting a balanced lifestyle that supports physiological and psychological health, thereby enhancing overall health. Personal well-being practices involve an approach that includes diverse strategies, dopamine roles, resilience, mindful awareness, supportive relationships, and self-care. This approach promotes a balanced, fulfilling life by encompassing physical, emotional, and psychological dimensions [30]. To enhance your well-being, consider incorporating alternative methods like nature-time, creative expression, or community service. Diversifying your well-being toolkit can provide a variety of strategies that align with your individual preferences and contribute to a balanced lifestyle [29]. Holistic self-care involves nurturing physical, emotional, and mental well-being through activities like meditation, massage, aromatherapy, and hobbies. It promotes a deeper connection with oneself and enhances overall well-being. Practices like tai chi, qigong, and acupuncture balance physical and mental health [28,29]. Lifelong learning and exploration of psychology, neuroscience, and well-being sciences can further inform one’s approach to dopamine fasting and overall well-being [30]. Integrating additional elements into personalized well-being practices makes the approach more tailor-based and the effect stronger while allowing the use chance to adapt and evolve the strategy.

Exploring Mindfulness

Mindfulness meditation and stress reduction techniques enhance psychological resilience and well-being. Meditation and body scan exercises increase awareness of the present moment, while progressive muscle relaxation and guided imagery help manage stress and promote overall well-being [31]. Immersing yourself in nature can serve as a source of profound rejuvenation and well-being [32]. Spending time outdoors, whether through nature walks, gardening, or simply enjoying nature [31]. By exploring and harnessing the power of nature, you can further enhance your well-being journey and find solace amidst the complexity of modern living. Incorporating mindfulness into daily rituals, such as mindful eating, breathing exercises, and gratitude journaling, can bring attention to the present moment and enhance overall well-being [33]. Mindful practices encourage a greater sense of awareness and appreciation for the experiences that shape daily life, fostering an outlook of mindfulness that aligns with the principles of dopamine fasting and wellness [34]. Holistic wellness involves incorporating practices like nature connection and mindfulness into daily routines, fostering social connections through meaningful conversations, group activities, and volunteering, and nurturing community relationships to enhance well-being and create a supportive environment [35]. Creative activities like art, writing, and music can improve a person’s well-being by providing emotional release, self-discovery, and a balanced approach. Nurturing emotional intelligence and self-reflection can enhance personalized practices, guiding the refinement of well-being strategies for each individual [31].

Nurturing Personal Growth and Reflection

Nurturing personal growth and self-reflection is essential for a holistic approach to well-being. Taking time for introspection, setting personal goals, and evaluating one’s values and beliefs supports continual self-improvement and a deeper understanding of oneself. Embracing personal growth encourages individuals to adapt to life’s challenges and evolve in alignment with their core values. By incorporating these dimensions of intellectual growth, creativity, and personal development into your well-being practices, you can further enhance your journey toward well-being and cultivate a fulfilling life. As you continue your journey toward well-being, it is essential to embrace a comprehensive approach in your daily life. By integrating various practices and principles that encompass physical, emotional, social, and intellectual dimensions, you can cultivate a balanced and fulfilling life [33]. Embracing mindful eating goes beyond the types of food you consume; it involves being present and attentive while eating, savoring each bite, and paying attention to hunger and fullness cues. By incorporating mindfulness into your eating habits, you can enhance your relationship with food, promote healthier digestion, and cultivate a more conscious and holistic approach to nutrition. Additionally, prioritizing whole, unprocessed foods and considering the environmental impact of your food choices can further contribute to a comprehensive approach to holistic nutrition [29,34]. Incorporating holistic nutrition and physical activity into your lifestyle is vital for promoting overall well-being [32]. Paying attention to nourishing your body with a variety of whole foods, staying hydrated, and engaging in regular exercise supports your physical health and vitality. Whether it is through mindful eating practices, cooking nutritious meals, or participating in physical activities that you enjoy, prioritizing nutrition and movement contributes to a better approach to well-being [36].

Creating a Supportive and Mindful Living Environment

Creating an environment that supports your well-being is essential for maintaining balance and harmony in daily life. This can involve decluttering your living space, incorporating elements of nature into your home, and fostering a calming atmosphere. Additionally, practicing mindfulness in your daily routines, from the way you prepare your meals to how you engage with technology, contributes to a mindful and supportive living environment that nurtures your well-being. By incorporating these holistic approaches into your daily life, you can further enrich your journey toward well-being and cultivate a balanced and fulfilling life that aligns with your values and aspirations [37]. In addition to fostering social connections and community engagement, prioritizing a deep connection with nature can significantly contribute to well-being. Spending time outdoors, immersing yourself in natural surroundings, and engaging in activities such as hiking, gardening, or simply sitting in a peaceful natural setting can have profound effects on your overall well-being [38]. Connecting with nature allows you to experience a sense of tranquility and awe, fostering a deeper appreciation for the world around you and promoting an overall sense of calm and balance [33]. Incorporating mindful movement practices such as yoga, tai chi, or qigong into your exercise routine can not only benefit physical health but also enhance overall well-being. These practices emphasize the connection between movement and breath, promoting a sense of mindfulness, body awareness, and relaxation [35]. Additionally, prioritizing regular physical activity that aligns with your interests and brings you joy can further support your well-being journey. By integrating these additional dimensions of well-being into your daily life, you can further enrich your journey and cultivate a balanced and fulfilling life that aligns with the principles of wellness [36]. Cultivating emotional resilience is an integral part of well-being. Acknowledging and managing your emotions healthily and constructively can have a profound impact on your overall well-being. Taking time for self-reflection, practicing mindfulness, and seeking support from trusted individuals can help you navigate through life’s challenges with greater resilience and emotional balance. Embracing positive affirmations and cultivating a gratitude practice can also contribute to your emotional well-being and enhance your overall sense of fulfillment.

Prioritizing Rest and Restoration

Prioritizing rest and restoration is integral to maintaining holistic well-being. Embracing practices such as adequate sleep, relaxation techniques, and mindful rest periods allows for physical and mental rejuvenation. By honoring the need for rest, you can enhance resilience, promote mental clarity, and support overall well-being. Prioritizing rest and quality sleep is essential for nurturing holistic well-being [33]. Establishing a consistent sleep routine, creating a relaxing bedtime environment, and incorporating relaxation techniques can support better sleep quality. Adequate rest and sleep are crucial for cognitive function, emotional regulation, and overall physical health, ultimately contributing to a more balanced and fulfilling life [37].

Engaging in Purposeful Work and Contribution

Engaging in work or activities that align with your values and offer a sense of purpose can significantly enhance your well-being. Whether through professional endeavors, volunteer work, or creative pursuits, finding meaning in your daily activities and contributing to something larger than yourself fosters a profound sense of fulfillment and overall well-being [38]. By integrating these dimensions of emotional resilience, rest, meaningful relationships, and purposeful work into your well-being journey, you can further enrich your life and cultivate a balanced and fulfilling existence that aligns with your values and aspirations. Exploring holistic approaches to mental well-being involves nurturing your mind and spirit through various practices. Incorporating mindfulness meditation, journaling, or engaging in creative activities can help in managing stress, improving mental clarity, and fostering emotional balance. These practices can provide a sense of inner peace and tranquility, contributing to a holistic approach to mental well-being [31]. Prioritizing personal growth and development is essential for well-being. Setting achievable goals, seeking opportunities for learning and self-improvement, and embracing challenges can lead to a sense of fulfillment and purpose. Continuously expanding your knowledge and skills contributes to a holistic approach to personal development, nurturing your overall well-being. Amidst the exploration of dopamine fasting and its potential impact, seeking professional guidance from healthcare providers or mental health professionals can provide personalized recommendations and support in developing an approach that aligns with individual needs and goals [17]. Engaging in open discussions and leveraging the expertise of professionals can offer valuable insights into the applicability of dopamine fasting in promoting psychological balance and overall well-being [18].

Critics of dopamine fasting

Dopamine Fasting is a new scientific ideology recognized by modern-day medicine but not accepted. It will be a long time if it provides enough scientific evidence to warrant its use in the places indicated by the said evidence but until then, modern-day medicine will deny the benefits of the ideology and frankly, without the evidence, they cannot and should not be stopped. The ideology and the techniques are proven by the generations which are long gone but the circumstances have changed and with that, the human mind as well. The benefits should outweigh the conditions of not using dopamine fasting to persuade scientists and allopaths to adopt the methods [37]. The techniques on the surface look similar to religious sacrifices of letting go of the stuff that makes a human happy and the implementation of it would be tough. Evolution has developed every living organism to act on reward-based mechanisms and the reward-punishment cycle has governed organisms through millions of years and rounds of evolutions. From a small amoeba going toward a food particle to Pavlov’s dog experiment, the entire history of life has been based on a reward system. [39,40,41]. Humans are no different than these organisms as at the end of the day, we are all products of evolution developing the brain from those primitive reward-based systems only [18,42]. Allopaths and modern-day medicine inherently do not believe in religious extremist activities and sacrifices of self. It is tough to include an idea about selflessness and depriving oneself of happiness into an ideology that has the sole purpose of saving lives and making the lives better for the patients and happier as well. The implantation of dopamine fasting will be challenged by modern-day medicine as long as it does not provide any benefits to a patient in a current manner of time and the ongoing criticism will only increase without evidence and proof [18].

Future implications of dopamine fasting

Dopamine fasting might prove to be a useful tool in patients with addictive behaviors and obsessive-compulsive disorder (OCD). It can be used in the treatment of impulsive behaviors and personality disorders. These theoretical possibilities are engaging as the current treatment protocol includes medicines that might become harmful in the end [4-6]. This beneficial role is yet theoretical. Proper evidence of it working in ideal and then non-ideal research conditions with results proving to have a significant difference in the general population will be required to have the ideas of the dopamine fasting technique implemented in the current guidelines for treating these diseases [18]. Although not from a medicinal point of view, this technique and ideology can prove to be a great and useful way for individuals seeking a more balanced and calmer lifestyle with insightful learnings about self and the surroundings to lead a peaceful and fulfilling individual-centric life. Implementation of the techniques in boundaries of human limits with the help of a qualified professional can be helpful and a blessing in the eternal circle of pain that is known as life.

## Conclusions

Dopamine fasting is a complex and evolving concept that requires careful consideration and mindful application. While it may offer benefits like reducing impulsivity and enhancing focus, it is essential to approach it with a balanced perspective and prioritize holistic well-being. This includes healthy lifestyle choices, regular physical activity, and a balanced diet to support overall brain health and neurotransmitter function. Incorporating emotional resilience, rest, meaningful relationships, purposeful work, and holistic approaches to mental well-being, and self-care practices into daily life is a powerful step toward nurturing holistic well-being. By embracing these approaches, individuals can cultivate a balanced and fulfilling life aligned with their values and aspirations. Prioritizing emotional resilience, rest, meaningful relationships, mental well-being, and self-care rituals contribute to overall fulfillment and well-being. Nurturing well-being is an ongoing journey, and by integrating these practices, individuals can foster a harmonious and enriched existence that supports their wellness aspirations. It is crucial to continue exploring and implementing these holistic approaches as you navigate life, always prioritizing your holistic well-being.
